# A Descriptive Study of Keel Bone Fractures in Hens and Roosters from Four Non-Commercial Laying Breeds Housed in Furnished Cages

**DOI:** 10.3390/ani10112192

**Published:** 2020-11-23

**Authors:** Käthe Elise Kittelsen, Randi Oppermann Moe, Tone Beate Hansen, Ingrid Toftaker, Jens Peter Christensen, Guro Vasdal

**Affiliations:** 1 Animalia—The Norwegian Meat and Poultry Research Centre, Lorenveien 38, NO-0585 Oslo, Norway; tone.beate.hansen@animalia.no (T.B.H.); guro.vasdal@animalia.no (G.V.); 2 Faculty of Veterinary Medicine, NMBU—Norwegian University of Life Sciences, PO Box 8146 dep., NO-0033 Oslo, Norway; randi.moe@nmbu.no (R.O.M.); ingrid.toftaker@nmbu.no (I.T.); 3 Department of Veterinary & Animal Sciences, University of Copenhagen, 1165 Copenhagen, Denmark; jpch@sund.ku.dk

**Keywords:** keel bone fracture, laying hen, animal welfare, poultry welfare, gene preservation

## Abstract

**Simple Summary:**

The keel bone in birds is an extension of the sternum. Fractures to the keel are common in modern laying hen breeds. Several of the proposed causal mechanisms behind keel bone fractures (KBF) are linked to selection for efficient production. It is, therefore, of interest to explore whether less selected breeds have a lower occurrence of keel bone fractures compared to reports from highly selected, modern laying hen breeds. Thus, the aim of the current study was to investigate keel bones of hens from four non-commercial layer breeds. Birds were housed in furnished cages and keel bones examined at 30 and 63 weeks of age using a portable X-ray equipment. The results from this descriptive study indicate a low prevalence of keel bone fractures in hens at both ages in all four breeds. No fractures were observed in the examined roosters. The overall low numbers of fractures indicate that genetic factors may be involved and, thus that selective breeding may help to reduce the susceptibility to keel bone fractures. Finally, this study highlights the importance of poultry conservation to secure existing genetic diversity, which may be an important resource in future selection schemes.

**Abstract:**

The presence of keel bone fractures (KBF) in laying hens has been documented and discussed by several authors, nevertheless the causative factors behind KBF remain uncertain. High prevalence of KBF have been reported in all commercial egg production systems, in different genetic lines and at different ages. Several of the proposed causal mechanisms behind KBF are linked to selection for efficient production. It is, therefore, of interest to explore whether less selected breeds have a lower occurrence of keel bone fractures compared to reports from highly selected, modern laying hen breeds. Thus, the aim of the current study was to investigate keel bones of hens from four non-commercial layer breeds. Birds were housed in furnished cages and keel bones examined at 30 and 63 weeks of age, using a portable X-ray equipment. The results from this descriptive study indicate a low prevalence of KBF at both ages in all four breeds, with only five KBF detected in 213 X-ray pictures taken from 126 birds. Of these, four of the KBF were observed in the most genetically selected breed, with an early onset of lay. None of the roosters examined exhibited KBF. The overall low numbers of KBF found indicate that genetic factors may be involved in KBF and, thus that selective breeding may help to reduce the susceptibility to KBF. Finally, this study highlights the importance of poultry conservation to secure genetic diversity, which may be an important resource in future selection schemes.

## 1. Introduction

Bones have two major functions in avian species: as a reservoir for calcium and phosphorous and as a support for the musculature [1]. The bone quality of modern laying hens has been a topic for scientific research since the 1950s, focusing mainly on osteoporosis [2,3,4,5]. A growing concern affecting avian bones is fractures to the keel bone [6,7,8], which has gained increasingly more attention the last decade.

Keel bone fractures (KBF) have been defined as fragmentation, shearing, or bending of the keel bone [7]. The prevalence of KBF in modern laying hens in commercial production systems is alarmingly high, reported higher than 80% in some studies [9,10,11,12,13]. Several fractures of the keel in the same bird is not uncommon [14]. In contrast, a recent pilot study of the ancestor of modern layers, the red jungle fowl (*Gallus gallus gallus*), found a much lower prevalence [15]. KBF have been found to a varying degree in all major commercial production systems, barren and furnished cages, non-cage systems and organic production [11]. Compared to cage systems, the prevalence is found to be higher in loose-housed systems [12,16,17]. Studies investigating the occurrence of KBF in roosters are generally lacking, but one study reported no cases of KBF in the included roosters [16]. Welfare implications of KBF include the associated pain [18,19,20], reduced mobility [21,22,23] and altered affective state [24]. The high prevalence, along with the impact on affected animals, makes KBF one of the biggest welfare challenges faced by the laying hen industry today [25]. In addition to welfare consequences, KBF have been found to be associated with reduced laying performance [23].

Despite extensive scientific effort over the last decade, the etiology of KBF is still not clear. It seems likely that KBF is a multifactorial disorder [26,27]. Several risk factors have been suggested: trauma and fractures due to high impact collision with the elements in the housing system [28], selection for increased egg production [16], hen age [23], osteoporosis [29,30], early onset of lay [31,32], and late ossification of the keel [14,27]. Interestingly, hens treated with hormones to suppress egg laying have been shown to have a lower risk of KBF [16,33]. Keel bone investigation in different strains and lines of modern layer hybrids indicate that genetic lines differ in the prevalence of KBF [6,8,9,30,34]. The cause of these differences between hybrids is uncertain; however, selection for high laying performance may be one important factor [16]. On the other side, selection for other traits, such as bone strength, may reduce the risk of KBF [34]. Besides one investigation of keel bones from the red jungle fowl [15], reports on KBF in non-commercial laying hen breeds are lacking.

Different assessment methods can be used to evaluate keel bones. Palpation is the most common method used in live hens [35]. Palpation relies on detection of the callus formed during fracture healing [36]. However, callus takes some time to develop; it is estimated that healing time for keel bone fractures in laying hens is six weeks [25]. New fractures, fractures with little callus formation or small fractures may be difficult to palpate leading to a low accuracy of a diagnostic procedure consisting of palpation only [35,37,38,39]. Furthermore, mobile fracture sites will create more periosteal callus formation than fracture sites with less mobility [40]. Thus, palpation might result in a larger underestimation of KBF occurrence in caged hens compared to loose-housed birds with more activity [14]. To accurately determine the prevalence of KBF, dissection or radiographs are considered the most reliable methods [39,41].

Several of the proposed causal mechanisms behind KBF are linked to selection for efficient production. It is, therefore, of interest to explore whether less selected breeds have a lower occurrence of keel bone fractures compared to reports from highly selected, modern laying hen breeds. Thus, the aim of the current study was to investigate keel bones of hens from four non-commercial layer breeds at two different ages. Furthermore, we wanted to examine the keel bones of roosters from the same breeds and ages in the same holding.

## 2. Materials and Methods

This descriptive study included a convenience sample from four different laying hen breeds (Table 1). Birds of all breeds were hatched, reared, and housed at the Norwegian live poultry gene preservation bank at Hvam Agricultural College, Norway. These breeds and lines have been maintained, since 1995, by a rotational mating scheme [42]. The birds were raised in cages (120 × 49 × 54 cm, length × width × height) furnished with one long perch (120 cm) in the middle height of the cage, a nest box, and a dust bathing area (Modell T8, Victorsson Poultry AB, Frillesås, Sweden). Housing was identical during both rearing and production. Each cage housed one rooster and six hens. All birds were wing tagged with unique numbers.

Birds were radiographed at 30 and 63 weeks of age (WOA) to include information at which age the KBF occurs. At 30 WOA 112 birds from 16 cages were examined: 96 hens and 16 roosters (Table 2). At 63 WOA 101 birds were examined: 85 hens and 16 roosters (Table 2). Several hens were sold between the two examinations thus only 55 of the original 96 hens radiographed at 30 WOA could be examined at 63 WOA. Therefore 30 new hens from the same four breeds were included at the second investigation.

The non-anesthetized birds were gently held upside down by a grip in both legs, inducing immobility. The left side of the bird was facing the digital flat panel detector and the keel bone was at a right angle. Digital radiographs were taken using a portable radiograph unit (Konica Minolta, Aero DR NS3543 mobil) with images obtained using a Poskom Vet20-BT. Training in X-ray handling and evaluation was received from Medivet Scandinavian AB, Ängelholm, Sweden. Images were taken with 50.0 kV, 2 mAs and a focus-film distance of 100 cm. All radiographic images were scored by the same person for the absence (0) or presence of one or more (1) keel bone fractures.

This study comprised non-invasive radiographic examination of keel bones of laying hens and roosters. Therefore, approval by an ethics committee for animal experiments was not required according to Norwegian legislation [45].

## 3. Results

In the present study, a total of 126 hens were radiographed, at both 30 and 63 WOA. In addition, 16 roosters were radiographed, all 16 at both occasions resulting in 213 radiographs in total. At 30 WOA, three hens were classified with KBF; two (8.3%) of the 24 hens from the Norbrid 8 breed and one (4.2%) of the 24 hens of the Minorca breed (Table 2). Both hens had a single fracture in the caudal third of the keel bone. The Minorca hen with fracture at 30 WOA had been sold and could not be investigated a second time at 63 WOA. At 63 WOA, two hens were classified with KBF both from the Norbid 8 breed. One of these had multiple fractures affecting both the middle and the caudal part of the keel; this bird was diagnosed with new fractures at both 30 and 63 WOA. The other had a single fracture in the caudal third of the keel; No fractures were observed in the roosters at any age.

**Figure 1:**
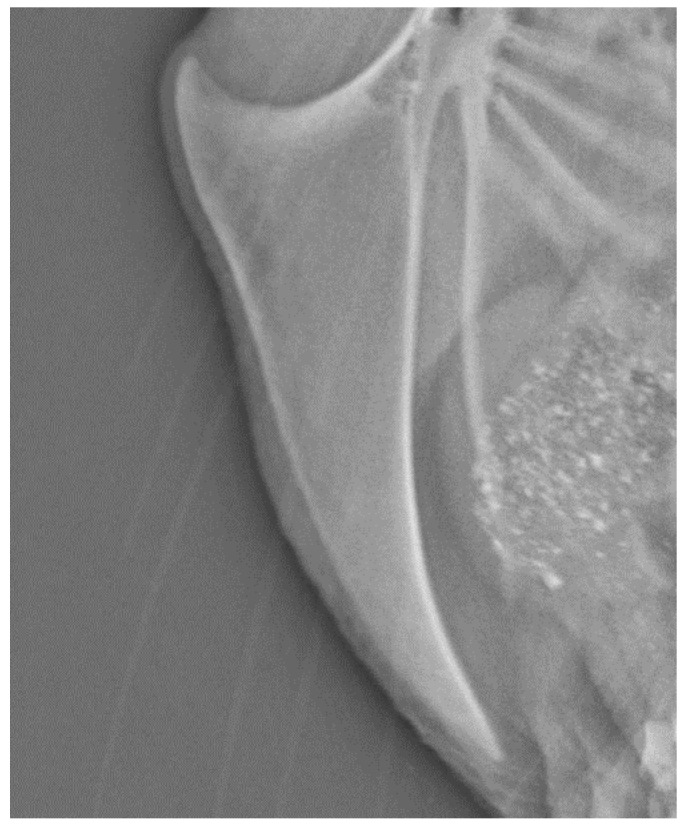
Normal, unfractured keel bone, 63 weeks of age.

**Figure 2:**
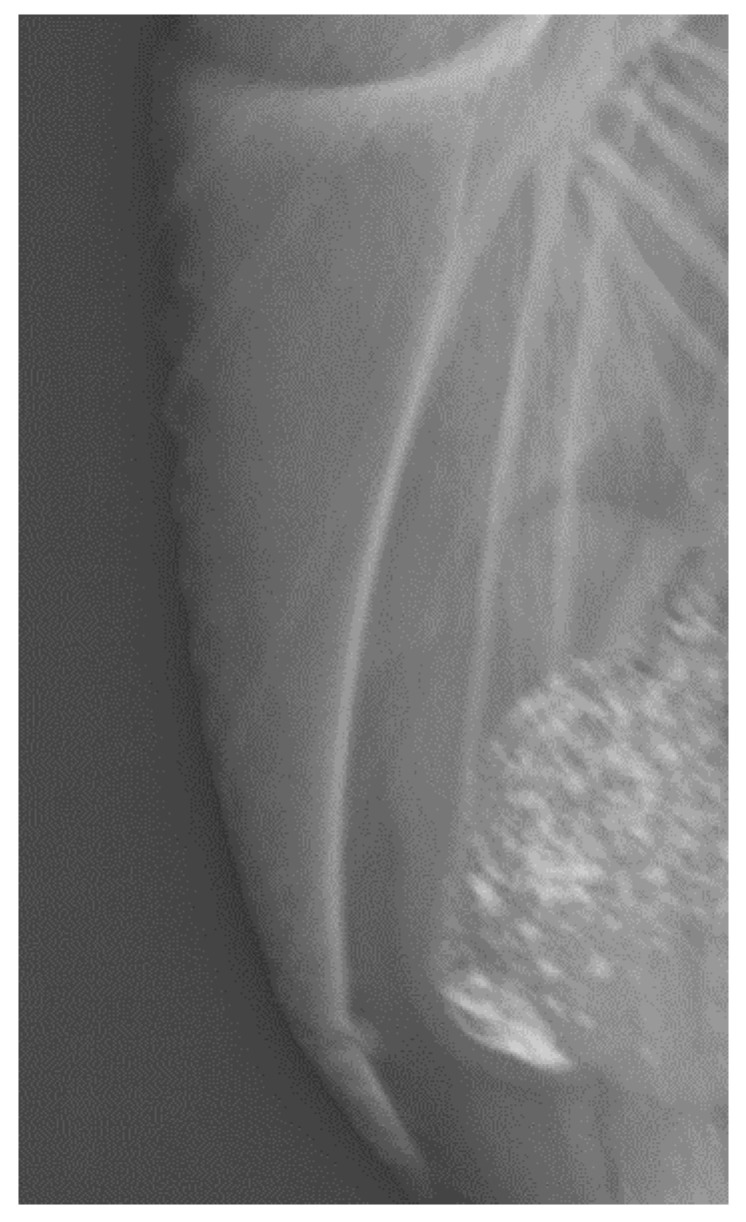
A keel bone with one fracture, 30 weeks of age.

**Figure 3:**
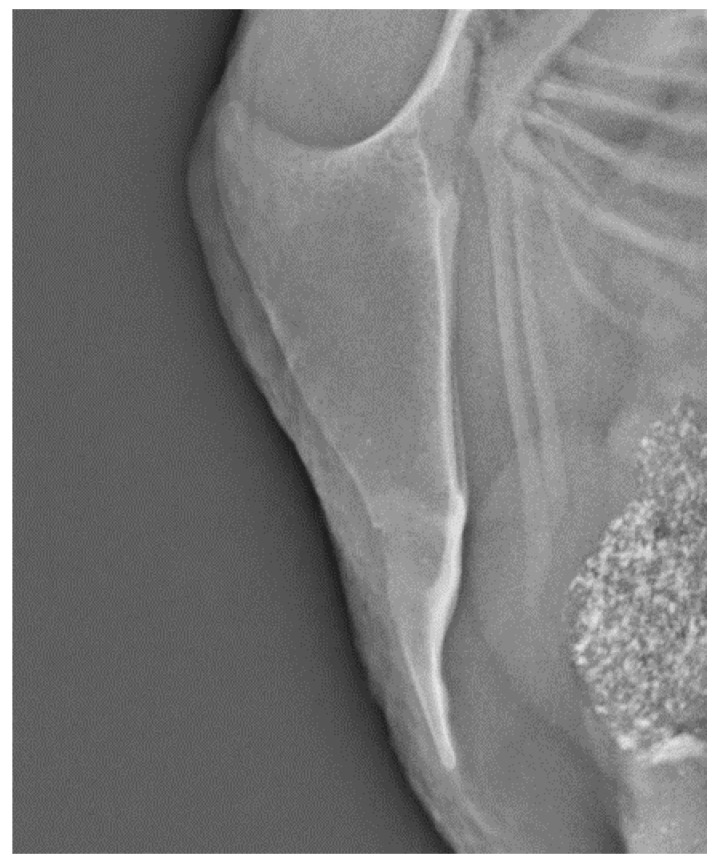
A keel bone with multiple fractures, age 63 weeks of age.

All fractures were located at the middle or caudal part of the keel bone. Figure 1 illustrates a normal keel bone without fracture. Keel bones with fracture are characterized by fragmentation or bending (Figure 2 and Figure 3). 

## 4. Discussion

This study used portable radiography to examine keel bones of hens and roosters from four non-commercial layer breeds at 30 and 63 weeks of age in order to explore whether less selected breeds have a lower occurrence of keel bone fractures compared to reports from highly selected, modern laying hen breeds. Overall, the prevalence of KBF was low among all breeds investigated. None of the investigated birds from the breeds Islandic landrace and Roko exhibited keel bone fractures, a result in accordance with findings in the red jungle fowl, which is the ancestor to all laying hen breeds [15]. In the Minorca breed one animal exhibited a fracture. The highest number of KBF was found in the breed Norbrid 8, with 2 KBF out of 24 (8.3%) examined birds at 30 WOA and 2 KBF out of 20 examined birds (10%) at 63 WOA. The Norbrid 8 is the most modern and selected of the four breeds included in the study. This breed was used as the male line in the Norwegian commercial laying hen breeding program until 1994, when it was replaced by international laying hen breeds. The low occurrence stands in contrast to published results from modern layer breeds, ranging from 30-97% [9,11,13]. It must be noted that comparison of KBF occurrence across studies can be challenging due to the sensitivity of different assessment methods [41]. However, an association between breed and KBF occurrence has been found in previous studies reporting various prevalence of KBF in different strains and lines of modern layer breeds [6,7,34]. Selection for a high laying performance may be one important factor that contribute to the different prevalences between hybrids [16]. This fits well for the breeds included in the current study, since they have not been subjected to the same selection. The strength of laying hen’s bones, which may be a risk facture for development of KBF, has also been found to vary between lines [34]. Therefore, including robustness toward KBF development, such as increased bone strength, in multi-trait selection program could be an important preventive measure to reduce the occurrence of KBF in layers, and thus improve laying hen welfare.

Age of first egg (AOF) varied from 15 to 22 weeks in the breeds in this study. Onset of lay is a parameter, such as high egg production, affected by genetics and selection [27]. The breed with the earliest AOF coincided with the breed with highest prevalence of KBF. This finding is in agreement with Andersson et al. (2017) who found early egg numbers to be associated with KBF in modern layer lines [31,32], where AOF is typically around 16 WOA. However, Roko also had an early AOF, without any KBF. It is not clear why an early onset of lay that may predispose for KBF. It has been speculated that late ossification of the caudal part of the keel bone combined with an early AOF may contribute to KBF [27]. The ossification process of the keel bone has never been investigated in breeds included in the study, therefore we do not how this process is affected by selection. Future studies are needed to investigate the effect of production traits such as AOF, ossification, hen weight and egg weight on hen level.

The prevalence of KBF in the present study was low at both 30 and 63 WOA. This contrasts with several studies where the prevalence of KBF increases significantly with age [8,11]. However, the identified association between hen age and susceptibility to KBF in previous studies may be confounded by strain, susceptibility to KBF or sampling bias [23]. A weakness of the present study is the low number of birds per breed and the high number of birds that were not used further during the second examination. To assess the incidence of KBF during the entire production period, large scale longitudinal studies using non-commercial breeds are needed.

All hens in this study lived in identical enriched cages, a housing form that has been assumed to have fewer KBF than loose house systems like aviaries [23]. However, KBF in caged hens may have been underestimated in previous studies based on palpation, since they develop less callus [14]. To avoid this inaccuracy the current study used radiography to investigate the keel bones. Due to identical housing, this factor cannot explain the differences between the breeds in the current study. There are no published studies on KBF in these breeds housed in non-cage systems, therefore we do not know if housing is a factor that may affect the results. All the fractured keel bones had fracture sites dorsally in the caudal third of the keel. This anatomical location is in accordance with findings by both Thøfner et al. [14] and Bauer et al. [13]. Fractures in this location are difficult to detect by palpation, hence, these fractures contribute to the low accuracy and reliability of palpation [37]. This is of particular importance when comparing results from different studies, especially based on palpation. Another aim in the current study was to investigate prevalence of keel bone fractures in roosters. The examination revealed that none of the roosters displayed any keel bone fracture at either 30 or 63 WOA. This is in accordance with Fleming et al. (2004), one of the few studies that previously investigated keel bones from roosters [30]. It is also in accordance with findings in red jungle fowl roosters [15]. The lack of KBF in male specimens supports the speculation that KBF is linked to egg laying [16,33]; however further studies are needed to examine keel bones in roosters since the literature is sparse.

## 5. Conclusions

In the current study portable radiography was used to investigate keel bones of hens and roosters from four pure breed non-commercial layer breeds at 30 and 63 weeks of age. The findings indicate a low prevalence of KBF in the laying hens. Of the five KBF found in the 213 X-rays from 126 hens, four radiographs indicating KBF were from three hens of the same breed, which is the most selected and efficient breed in the study. None of the roosters examined exhibited KBF. The results indicate that selective breeding could reduce the susceptibility to keel bone fractures. Furthermore, the results from this study highlight the importance of poultry conservation to secure genetic diversity, which may be a genetic resource in future production and selection efforts.

## Figures and Tables

**Table 1:** Laying hen breeds and characteristics.

Breed	Classification	Origin ^1^	Onset of Lay, in Weeks ^2^
Icelandic landrace	Egg layer	The native breed of Iceland, originating from Old Norwegian Jadar ^3^. Not cultivated for specific characteristics	22
NorBrid 8	Egg layer	The paternal line of the last Norwegian, commercial layer hybrid, NorBrid 87. Descends from Red Rhode Island	15
Minorca	Egg layer	Developed in England from imported Castilian fowl	22
Roko	Egg layer	The oldest existing purebred line in Norway. Originated from White Leghorn ^4^	16

^1^ Information on origin is based on literature review; ^2^ Personal communication from Mette Nafstad Bjerkestrand, the Norwegian live poultry gene preservation bank, Hvam Agricultural College; ^3^ Data from Lyimo et al. 2014 [43]; ^4^ Data from Brekke et al. 2017 [44].

**Table 2:** Number of keel bone fractures depending on breed, sex and age (*n* = 213 ^1^).

Breed	Examination, 30 Weeks of Age				Examination, 63 Weeks of Age			
	Females	Females with Fractures		Roosters *	Females	Females with Fractures ^2^		Roosters *
	*n*	*n*	%	*n*	*n* ^3^	*n*	%	*n*
Icelandic landrace	24	0	0	4	19 ^4^	0	0	4
Norbrid 8	24	2	8.3	4	20 ^5^	2	10	4
Minorca	24	1	4.2	4	29 ^6^	0	0	4
Roko	24	0	0	4	17 ^7^	0	0	4
Total	96	3	3.1	16	85	2	2.4	16

* No fractures were detected in any of the roosters; ^1^ *n* = 213 X-rays, from 126 unique hens and roosters, some were repeated measures on the same birds; ^2^ One of the Norbrid 8 birds with fracture at 63 WOA was also diagnosed with a new fracture at 30 WOA; ^3^ 55 of these hens were X-rayed at 30 WOA and 30 hens were X-rayed for the first time at 63 WOA; ^4^ 9 hens X-rayed for the first time; ^5^ 5 hens X-rayed for the first time; ^6^ 10 hens X-rayed for the first time; ^7^ 6 hens X-rayed for the first time.
